# SPECT Imaging of Muscle Injury with [^99m^Tc]MDP in a Mouse Model of Muscular Dystrophy

**DOI:** 10.1007/s11307-019-01394-7

**Published:** 2019-07-08

**Authors:** Carolynn Gaut, Jenna M. Sullivan, Barbara Biscaro, Edward J. Soares, Katharine Nicholson, Jack Hoppin, Ajay Verma

**Affiliations:** 1 Invicro, Boston, MA USA; 2 Massachusetts General Hospital, Boston, MA USA; 3 United Neuroscience, Dublin, Ireland

**Keywords:** Duchenne muscular dystrophy, *mdx*, [^99m^Tc]MDP, Muscle calcification, SPECT, Imaging, Neuromuscular disease, Ectopic calcification

## Abstract

**Purpose:**

Tc-99m methylene diphosphonate ([^99m^Tc]MDP) is an *in vivo* bone imaging agent that also accumulates in injured skeletal muscle cells. The objective of this study was to investigate if [^99m^Tc]MDP could be used to detect muscle injury in the *mdx* mouse model of Duchenne muscular dystrophy (DMD).

**Procedures:**

Static whole-body single-photon emission computed tomography/computed tomography (CT) scans were acquired at 2 h post-injection of [^99m^Tc]MDP in two cohorts of animals at different sites: one cohort of mice at 6, 15, and 19 weeks of age, and a separate cohort at 16 weeks. The second cohort was also imaged with high-resolution CT at 8 weeks.

**Results:**

*mdx* mice had higher [^99m^Tc]MDP uptake and significantly higher [^99m^Tc]MDP concentrations in muscle than controls.

**Conclusions:**

Higher uptake of [^99m^Tc]MDP in muscle of *mdx* mice agrees with histological reports of muscle calcification in *mdx* mice, and suggests the potential translational use of [^99m^Tc]MDP imaging for tracking DMD progression and therapeutic response.

**Electronic supplementary material:**

The online version of this article (10.1007/s11307-019-01394-7) contains supplementary material, which is available to authorized users.

## Introduction

Duchenne muscular dystrophy (DMD) is a severe neuromuscular disease that affects 1 in 3500 boys and is characterized by progressive degeneration of striated muscle cells resulting from mutations in or loss of the cytoskeletal protein dystrophin [1]. This leads to sarcolemmal membrane injury, intracellular calcium elevation, muscle cell death, fibrosis, weakness, and loss of independent ambulation by the age of 13 years [2, 3]. Several therapeutic strategies are being investigated for DMD including corticosteroids, antioxidants, nutritional modification, calcium channel modifiers, exon skipping antisense RNAs, and gene therapies; however, the paucity of convenient, quantitative, and objective disease progression biomarkers that accurately reflect muscle cell injury has slowed the development and approval of such therapies [4].

The *mdx* mouse is a naturally occurring mouse model for DMD caused by deficiency of dystrophin, with a resulting moderate myopathology as compared with the human disease course. The myopathic phenotype starts around 3 weeks with muscle necrosis and some visible muscle weakness. The structure of neuromuscular junctions in *mdx* mice is also altered and by approximately 4 weeks of age, there is evidence of mild fibrosis and phagocytosis of necrotic muscle tissue [5–7] as well as degenerative lesions with calcification [7, 8]. Several medical imaging approaches have been used to assess a variety of disease-related processes in dystrophic mouse models, including changes in muscle architecture [7], sarcolemmal permeability [9], cardiac metabolic differences [10], and skeletal muscle degeneration and regeneration [11]. To date, however, there is no effective method to reliably, quantitatively, and non-invasively assess degenerative changes that occur as DMD progresses. There is also a clear unmet need to establish preclinically accurate evaluations of therapies for the treatment of muscle disease.

We hypothesized that the abnormal muscle cell calcification processes could be imaged using 99mTc-methylene diphosphonate ([^99m^Tc]MDP), a commonly used clinical radiotracer for bone and calcification substrates. Uptake of [^99m^Tc]MDP has been reported clinically in skeletal muscle due to ossification, injury, and necrosis [12]. The objective of this study was to assess [^99m^Tc]MDP uptake in the muscle of *mdx* mice at multiple time points during disease progression.

## Materials and Methods

### Animal Model

Male C57BL/10ScSn-Dmd^*mdx*^/J mice (*n* = 10, JAX no. 001801) and age-matched male C57BL/10ScSnJ mice (*n* = 10, JAX no. 000476) were purchased from Jackson Laboratories. The C57BL/10ScSn-Dmd^*mdx*^/J mouse is a model of DMD. Animals were acclimated for a minimum of 3 days prior to imaging. Animals were housed in pairs except after imaging when animals were single-housed to allow for radiation decay. Rodent chow (Lab Diet® Certified Rodent Diet no. 5002, PMI Nutrition International, Inc.) and tap water were available *ad libitum*. All applicable institutional and/or national guidelines for the care and use of animals were followed. All study procedures were approved by the Institutional Animal Care and Use Committee of MPI Research (Mattawan, MI) or Invicro, LLC (Boston, MA).

### Image Acquisition

All single-photon emission computed tomography (SPECT) images were acquired on a NanoSPECT/CT™ (Bioscan, Inc., Washington, DC, USA) or NanoScan™ (Mediso, Budapest, Hungary). The energy window used for acquisition was 126.459–154.561 keV. Whole-body scans were 32 projections per axial field of view with approximately 60 s per projection. A 9-pinhole aperture (diameter of 2.5 mm) was used. Immediately following each SPECT scan, a 65-kVp computed tomography (CT) scan (180 projections per rotation at 0.5–1 s per projection) was acquired over 8–10 min. All image data were reconstructed using the ordered subsets expectation maximization (OSEM) algorithm available within the HiSPECT software into 0.2 × 0.2 × 0.2 mm voxels.

[^99m^Tc]MDP was purchased from Hot Shots Nuclear Medicine (Kalamazoo, MI, USA) for use at MPI Research and from Cardinal Health Nuclear Pharmacy (Cleveland, OH, USA) for use at Invicro Boston. One cohort of animals (*n* = 5 per group) was scanned at 6, 15, and 19 weeks of age at MPI Research. A second cohort of animals (*n* = 5 per group) was imaged with high-resolution whole-body CT at 8 weeks and with [^99m^Tc]MDP SPECT at 16 weeks of age at Invicro Boston. For SPECT/CT imaging, [^99m^Tc]MDP-specific activity per volume was greater than 12 mCi/ml and calibrated at 9:00 am on the day of imaging. Animals were weighed prior to imaging at each time point. [^99m^Tc]MDP was injected intravenously in awake animals. Tracer injection information can be found in Table 1. At 2 h post-[^99m^Tc]MDP injection, each animal was anesthetized with isoflurane and imaged by whole-body SPECT (1 × 30 min static scan) followed by CT.

**Table 1:** Injected dose summary of [^99m^Tc]MDP imaging

Animal age time point	Group and no. of animals	Animal weight (g)	Injected dose (μCi/animal)
6 weeks	*mdx* (*n* = 5)	18.9 ± 3.0*	629 ± 20
	Control (*n* = 5)	22.3 ± 1.1	629 ± 23
15 weeks	*mdx* (*n* = 5)	30.5 ± 1.4*	689 ± 90
	Control (*n* = 5)	27.8 ± 1.3	744 ± 19
19 weeks	*mdx* (*n* = 5)	31.7 ± 1.1*	760 ± 12
	Control (*n* = 5)	28.7 ± 1.2	756 ± 28
16 weeks^a^	*mdx* (*n* = 5)	27.4 ± 1.8*	691 ± 17
	Control (*n* = 5)	30.1 ± 1.2	706 ± 29

^a^Animals imaged at 16 weeks of age are a separate cohort than the animals imaged at 6, 15, and 19 weeks of age

*Body weight is significantly (*p* < 0.05) different between *mdx* and control animals at the time point indicated

Values shown are mean ± SD

Following the final *in vivo* imaging time point at 19 weeks of age, this cohort of animals was immediately sacrificed and perfused with phosphate buffered saline, and the diaphragm from each animal was resected. Although the limb muscle phenotype in the *mdx* mice is known to be rather mild, a recapitulation of the muscle degeneration and fibrotic process has been previously confirmed for the diaphragm muscle in these animals [13]. The diaphragm is difficult to image in live animals due to its thinness, apposition to the rib cage, and respiration-associated motion. To more clearly assess [^99m^Tc]MDP signal in the diaphragm, we imaged diaphragms *ex vivo* following post-mortem dissection. Each diaphragm was fixed in formalin gel and then positioned on the side of a 10-ml plastic syringe (four diaphragms per syringe at most). Diaphragms were imaged with SPECT (1 × 30 min) followed by high-resolution CT at approximately 4 h post-injection of [^99m^Tc]MDP.

For the whole-body high-resolution micro-CT image acquisition, animals were fasted at least 12 h prior to imaging and anesthetized with isoflurane for the imaging procedure, which was performed on the NanoScan™ Helical CT at a voltage of 70 kVp and a current of 310 μA (720 projections at 170 ms per projection) over 8–10 min. Medium zoom was applied with a 4:1 binning. All image data were reconstructed using the OSEM algorithm available within the HiSPECT software. Two Bruker phantoms were included in each scan and placed parallel to the *z*-axis near the head. Following imaging, animals were recovered and returned to the home cage.

### Data Analysis

To assess the uptake and distribution of [^99m^Tc]MDP, regions of interest (ROIs) corresponding to the skeleton, heart, kidneys, left quadriceps, right quadriceps, left scapular muscle, right scapular muscle, ribs, intercostal muscles, and diaphragm were defined on co-registered SPECT/CT images. The ROIs corresponding to the heart and kidneys were defined by fitting predefined phantoms of fixed volume to the regions on the SPECT/CT data. The left and right quadriceps’ ROIs were hand-drawn by placing spheres of ~ 10 mm^3^ in the quadriceps region of each leg. Similarly, the left and right scapular muscle ROIs were hand-drawn by placing spheres of ~ 10 mm^3^ in the scapular region of each shoulder. Fixed volume ROIs were used to eliminate any size bias from the analysis and the volumes were determined based on mouse anatomy. To segment the rib and intercostal muscle regions, a region encompassing the rib cage was defined manually based on CT for each animal; the sternum and thoracic vertebrae were excluded from this region. Ribs were segmented from the defined region using thresholding based on CT. A threshold value of 500 Hounsfield units (HU) was set for data from the cohort imaged at 6, 15, and 19 weeks and 1100 HU was set for the week 16 data. Voxels containing values above these thresholds were defined as “rib.” The remaining voxels within the region encompassing the rib cage were assigned as “intercostal” to represent intercostal muscle and tissue. The diaphragm ROI was defined by applying segmentation thresholds, determined based on lung anatomy, to the CT and dilated. The skeleton ROI was defined by applying segmentation thresholds to the CT data.

*Ex vivo* diaphragm ROIs were defined by applying several segmentation thresholds to the CT. The syringe was segmented and removed from the ROI and each diaphragm was segmented individually by creating boundary areas for each distinct diaphragms. Boundary areas that were not clearly belonging to an individual diaphragm and artifacts in the CT were manually removed from the ROI and not included in the analysis.

The concentration of tracer was extracted from each ROI and reported in standard uptake value (SUV), which is the concentration normalized by injected dose and animal weight. Whole-body high-resolution micro-CT images were segmented into ROIs identified as bone (voxel values > 700 HU) and ectopic calcification (300–700 HU) based on intensity. Image analysis was performed in VivoQuant™ version 2.50patch3 (Invicro, LLC, Boston, MA, USA) and MATLAB version 2013b (MathWorks, Natick, MA, USA).

### Statistical Analysis

For each organ, the values of [^99m^Tc]MDP SUV or CT-based volumes for the *mdx* and control groups were compared using unpaired *t* test with alpha of 0.05 and without correction for multiple comparisons. Statistical analysis and plotting were performed in Prism version 6.05 (GraphPad Software, La Jolla, CA, USA). Statistically significant results are indicated by one asterisk (*; *p* < 0.05), two asterisks (**; *p* < 0.01), or three asterisks (***; *p* < 0.001).

## Results

At all imaging time points, the control and *mdx* groups significantly (*p* < 0.05) differed in body weight (Table 1). At 6 weeks, *mdx* mice weighed significantly less than the controls, but the body weight in *mdx* mice increased over time and *mdx* mice weighed significantly more than the controls at 15, 16, and 19 weeks.

Of the 5 animals per group scanned at the 15-week time point, one *mdx* animal was removed from the analysis due to a poor [^99m^Tc]MDP injection. Of the 5 animals per group scanned at the 16-week time point, one control animal and one *mdx* animal were removed from the analysis due to abnormally high kidney signal. Kidney SUV from these animals at the 16-week time point was outlying as determined by the Grubbs’ test (*α* = 0.05, GraphPad Prism 7.02).

**Fig. 1.**
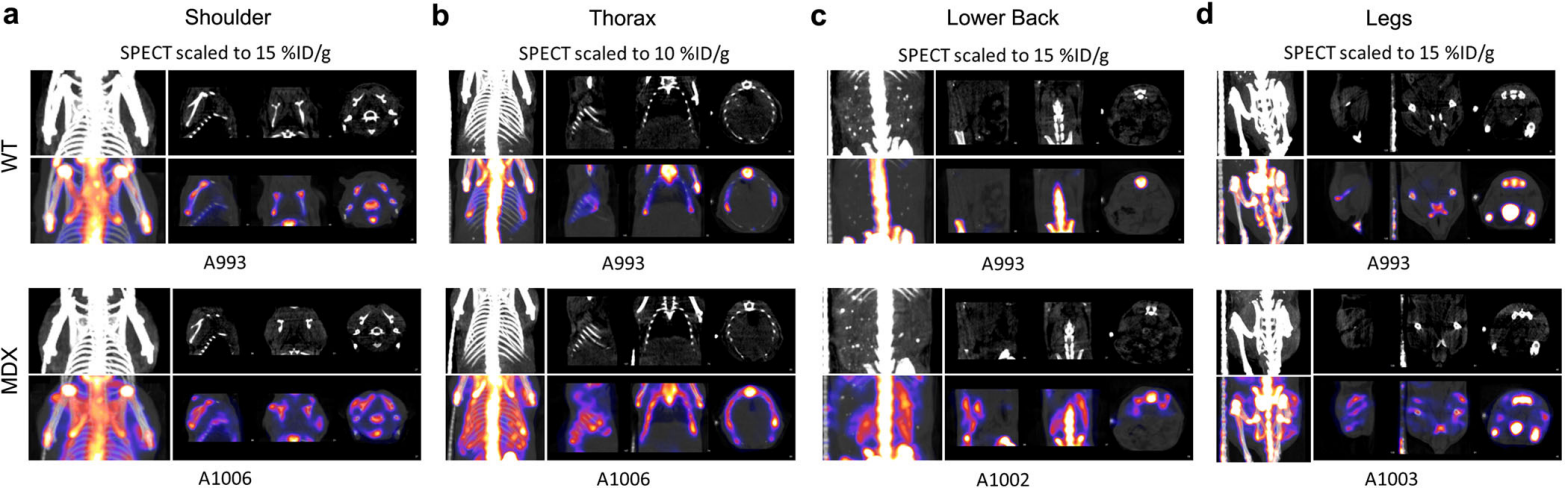
[^99m^Tc]MDP distribution at 2 h post-injection in the **a** shoulder, **b** thorax, **c** lower back, and **d** hind limbs of representative *mdx* and control mice at 6 weeks of age. SPECT scans are shown overlaid on CT (in grayscale) for anatomical reference (CT scaled to 0–1000 HU).

**Fig. 2.**
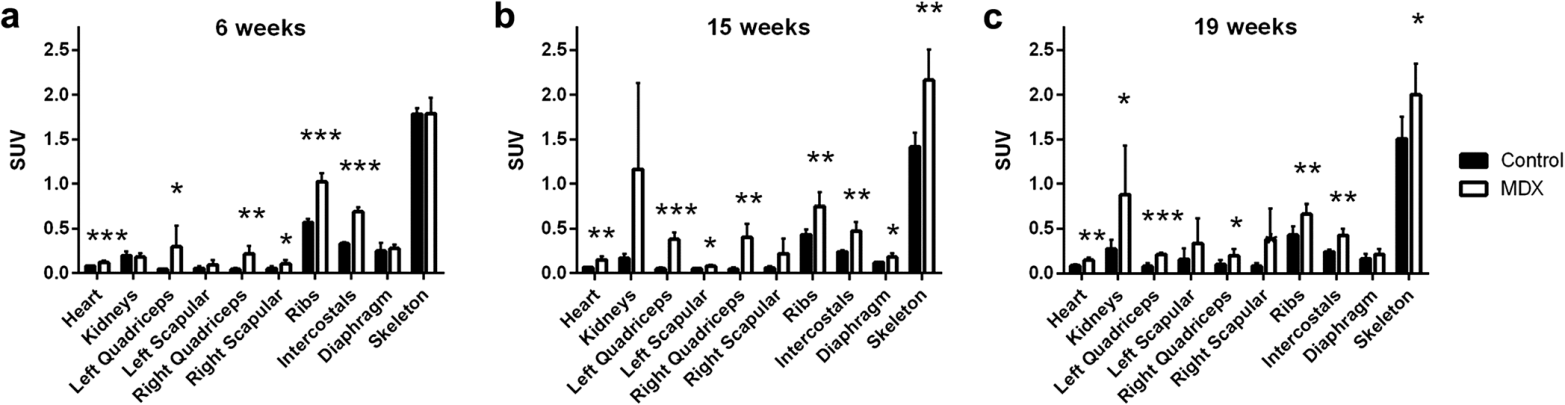
[^99m^Tc]MDP biodistribution in control and *mdx* mice at **a** 6 (*n* = 5 each group), **b** 15 (*n* = 5 control mice, *n* = 4 *mdx* mice), and **c** 19 (*n* = 5 each group) weeks of age. Tissue concentration in the heart, kidneys, left quadriceps, left scapular muscle, right quadriceps, right scapular muscle, ribs, intercostal muscles (intercostals), diaphragm, and skeleton is shown as standard uptake value (SUV). Bars are mean and error bars are standard deviation (SD). Values from the control animals are shown in black and values from the *mdx* animals are shown in white. Significant differences between the control and *mdx* animals are indicated by one asterisk (*; *p* < 0.05), two asterisks (**; *p* < 0.01), and three asterisks (***; *p* < 0.001). Of the original 5 animals per group scanned at the 15-week time point, one *mdx* animal was removed from the analysis due to abnormally high kidney signal.

At all imaging time points, *mdx* mice had higher [^99m^Tc]MDP uptake in muscle than the controls (see Electronic Supplementary Material). In the first cohort of animals, even at the earliest imaging time point of 6 weeks of age, *mdx* mice showed higher [^99m^Tc]MDP accumulation *versus* the control animals in several tissues including the heart, quadriceps, scapular muscles, ribs, and intercostal muscles (Fig. 1). These differences persisted through 15 weeks and 19 weeks as quantified in the bar graphs in Fig. 2. Higher [^99m^Tc]MDP uptake was observed in the muscles of the shoulder, thorax, lower back, and hind limbs of the *mdx* mice (Fig. 2) and was two to nine times higher in the *mdx* mice than the controls across the various muscle groups. In diaphragms imaged *ex vivo* from mice 19 weeks of age, *mdx* mice had significantly (*p* < 0.001) higher *ex vivo* [^99m^Tc]MDP SUV (0.240 ± 0.008) compared with the control animals (0.004 ± 0.001).

**Fig. 3.**
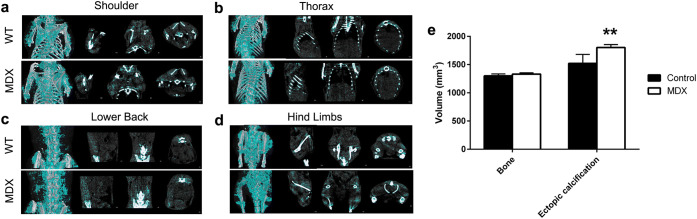
High-resolution CT-based segmentation of ectopic calcification in the **a** shoulder, **b** thorax, **c** lower back, and **d** hind limbs of the *mdx* and control mice at 8 weeks of age. Threshold-based segmentations of ectopic calcification are shown in teal, bone in white, and shown overlaid on CT (in grayscale) for anatomical reference. **e** Volume of bone and ectopic calcification segmented from high-resolution CT. Bars are mean and error bars are standard deviation (SD). Values from the control animals (*n* = 5) are shown in black and values from the *mdx* animals (*n* = 5) are shown in white. Significant differences between the control and *mdx* animals are indicated by two asterisks (**; *p* < 0.01).

**Fig. 4.**
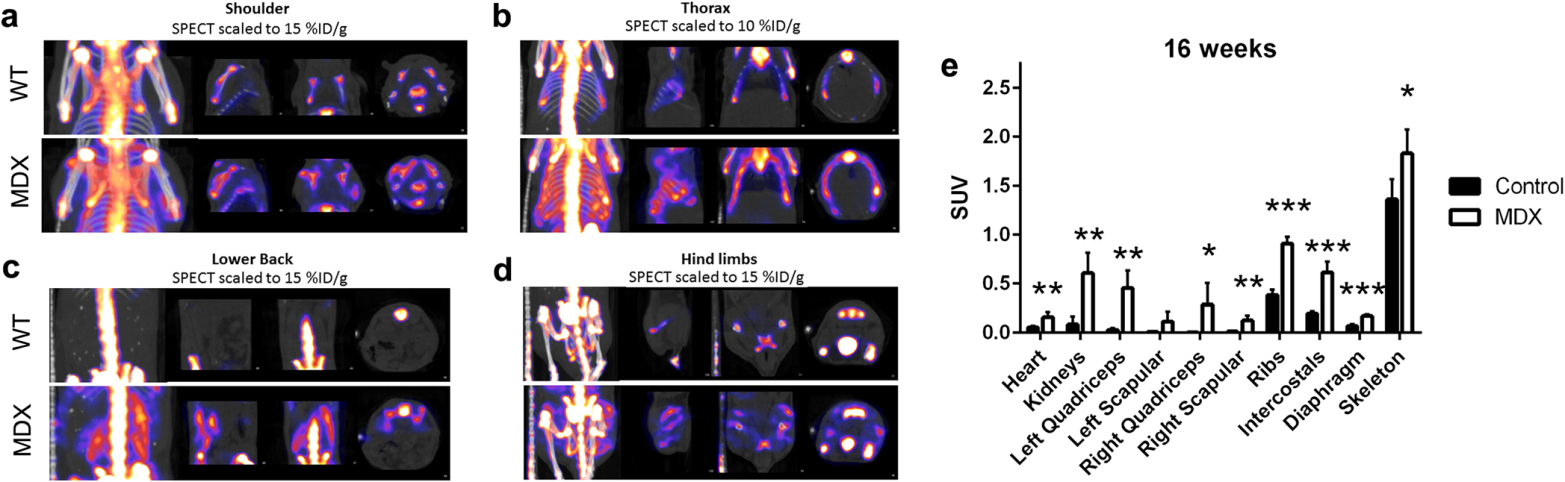
[^99m^Tc]MDP biodistribution in the control (*n* = 4) and *mdx* (*n* = 4) mice at 16 weeks of age. [^99m^Tc]MDP distribution at 2 h post-injection in the **a** shoulder, **b** thorax, **c** lower back, and **d** hind limbs of the *mdx* and control mice. SPECT scans are shown overlaid on CT (in grayscale) for anatomical reference. **e** Tissue concentration in the heart, kidneys, left quadriceps, left scapular muscle, right quadriceps, right scapular muscle, ribs, intercostal muscles (intercostals), diaphragm, and skeleton is shown as standard uptake value (SUV). Bars are mean and error bars are standard deviation (SD). Values from the control animals are shown in black and values from the *mdx* animals are shown in white. Significant differences between the control and *mdx* animals are indicated by one asterisk (*; *p* < 0.05), two asterisks (**; *p* < 0.01), and three asterisks (***; *p* < 0.001). Of the original 5 animals per group scanned at this time point, one control animal was removed from the analysis due to a poor [^99m^Tc]MDP injection and one *mdx* animal was removed from the analysis due to abnormally high kidney signal.

**Fig. 5.**
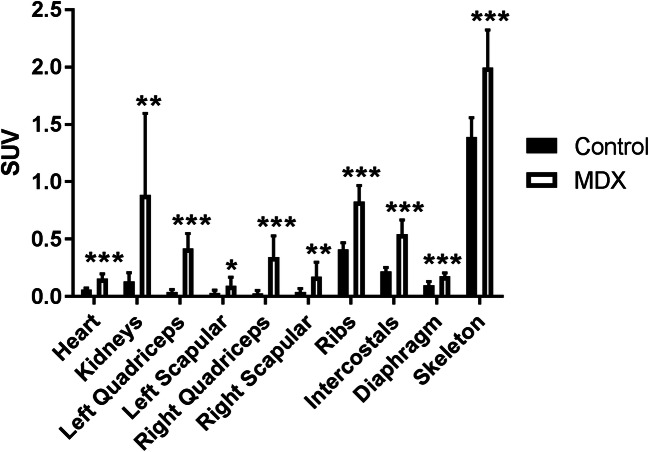
Pooled results from the control (*n* = 9) and *mdx* (*n* = 8) mice at 15 and 16 weeks of age. [^99m^Tc]MDP tissue concentration in the heart, kidneys, left quadriceps, left scapular muscle, right quadriceps, right scapular muscle, ribs, intercostal muscles (intercostals), diaphragm, and skeleton is shown as standard uptake value (SUV). Bars are mean and error bars are standard deviation (SD). Values from the control animals are shown in black and values from the *mdx* animals are shown in white. Significant differences between the control and *mdx* animals are indicated by one asterisk (*; *p* < 0.05), two asterisks (**; *p* < 0.01), and three asterisks (***; *p* < 0.001).

To assess the reproducibility of our findings, we repeated [^99m^Tc]MDP imaging in a separate cohort of animals at a distinct imaging site. In these animals, high-resolution CT and threshold-based segmentation images showed no obvious differences in non-bone signal (ectopic calcification) between groups; however, the total volume of ectopic calcification was significantly (*p* < 0.01) higher in the *mdx* mice than the controls (Fig. 3). There was no difference between the groups in the volume of voxels identified as bone. This cohort of animals was imaged with [^99m^Tc]MDP at 16 weeks to closely approximate the 15-week imaging time point of the first cohort and strikingly similar results were obtained both qualitatively and quantitatively (Fig. 4). When these data were pooled together with the 15-week data from the first cohort, a gain in statistical significance resulted from the increase in power (Fig. 5).

## Discussion

Using preclinical SPECT, we have found that the *mdx* mice have significantly greater uptake of [^99m^Tc]MDP in skeletal muscles than the age-matched healthy control animals. Higher [^99m^Tc]MDP signal in the *mdx* mice was seen in several muscle groups including those of the limbs (*e.g.*, quadriceps and scapular muscles), rib cage, and back (Figs. 1, 2, and 4). This pattern of high [^99m^Tc]MDP uptake in the *mdx* mice was apparent as early as 6 weeks and was consistent and statistically significant over the next 4 to 5 months. This is in agreement with prior histological studies performed in young (8 weeks) and old (24 months) *mdx* mice where skeletal muscle stained for heterotopic ossification with Alizarin red showed greater stain in the *mdx* mice compared with the control mice but no difference in staining between 8-week-old and 24-month-old *mdx* mice [14].

It is a limitation of this study that histological analysis of tissue nor motor function assessments were performed to compare the imaging results to measures of disease progression. The goal of this work was to assess the feasibility of [^99m^Tc]MDP to detect muscle injury in *mdx* mice, and emphasis was placed on methodology and analysis in this first-time application to enable subsequent explorations with this technique. Nonetheless, higher [^99m^Tc]MDP signal in *mdx* mice likely represents disease-related ectopic calcification in skeletal muscles. Sarcolemmal injury due to dystrophin mutations is known to increase the influx of calcium into striated muscle cells, which can form calcium deposits and lead to necrosis. Calcified deposits in the muscles of *mdx* mice have in fact previously been identified as hydroxyapatite, a crystalline form of calcium phosphate [7], and [^99m^Tc]MDP is known to strongly bind to hydroxyapatite, which is likely the underlying basis for the use of this radiotracer in routine bone scans [15]. Diphosphonates have been observed to accumulate in injured muscle tissue and thus may represent as a useful quantitative imaging biomarker for tracking muscle injury [16].

*mdx* mice have been shown to develop degeneration, inflammation, and fibrosis in the diaphragm muscle [17]. Although the [^99m^Tc]MDP signal in the diaphragm was difficult to evaluate in the *in vivo* SPECT data due to its close proximity to the rib cage and respiration-related motion, a significant (*p* < 0.001) difference between the *mdx* and control mice was detectable in the pooled 15- to 16-week data of the two cohorts. Additionally, when the diaphragms were excised and imaged *ex vivo*, a four times higher signal was seen in the *mdx* mice compared with the controls.

The heart of the *mdx* mice showed significantly (*p* < 0.05) greater uptake of [^99m^Tc]MDP than that of the controls. In addition to severe abnormalities in the skeletal muscle, DMD has also been shown to cause fibrosis and degeneration in cardiac tissue [18, 19]. Cardiac uptake of [^99m^Tc]MDP has been seen clinically in situations related to myocardial infarction where there is excess tissue calcium following local tissue damage or necrosis [12]. Recent histological evaluation of heterotopic ossification, however, did not find calcification in cardiac muscle of the *mdx* mice [14]. This may suggest that the higher [^99m^Tc]MDP signal in the heart of *mdx* mice may be related to general tissue damage rather than just calcification in the myocardium.

The primary route of clearance of [^99m^Tc]MDP is through the kidney, which may explain the high uptake seen in this region. It is unknown what mechanism underlies the significant (*p* < 0.05) difference in the kidney uptake between the groups, although recent macro- and microscopic examinations of kidneys in *mdx* mice found microhemorrhages and reduced urine spaces compared with the control animals [20]. Renal impairment is being increasingly recognized in patients with DMD [21, 22] and more detailed examination of renal histopathology in *mdx* mice is warranted in future studies.

High-resolution CT has been previously used to detect ectopic calcifications in *mdx* mice [23–25], which is accentuated by high-phosphorus diet. In our experiments in mice on a normal diet, we detected significant (*p* < 0.01) differences in ectopic calcification between *mdx* mice and controls with CT, and the pattern of the ectopic calcification agreed with that shown in a previous work [25]. The pattern of ectopic calcification measured by SPECT was much more striking than that seen with CT. In the CT data, signal defined as ectopic calcification was diffuse and noisy, and differences between *mdx* mice and controls were not as visually obvious. The high (micromolar to nanomolar) sensitivity of SPECT likely allows for the detection of disease-related calcification changes before the lesions become dense enough to be detected with CT. [^99m^Tc]MDP SPECT, therefore, may be more useful in translation to the clinic as an imaging tool in the treatment of DMD and other diseases where ectopic muscle calcification is expected.

## Conclusions

There is a clear need for non-invasive tools for diagnosis and monitoring of DMD disease progression as well as for screening potential therapies at the preclinical level. While eteplirsen was recently approved for this disorder, the approval process was challenged over the limited objective biomarker data supporting drug efficacy. Clinically translatable imaging biomarkers applicable to DMD disease processes could greatly support decision-making for future drug approvals. The *mdx* mouse model is often used for performing preclinical tests with the aim of identifying drugs able to target the pathogenic mechanisms to delay pathology progression [23]. We have shown that the *mdx* mouse model of DMD shows greater uptake of [^99m^Tc]MDP in muscle, particularly in the quadriceps and diaphragm, as compared with the controls. This agrees with histological reports of muscle calcification in *mdx* mice [8], the well-known target of diphosphonates to hydroxyapatite calcium deposits [15], and prior clinical observations of [^99m^Tc]MDP accumulation in injured or dystrophic skeletal muscle [16, 26, 27]. These results were consistent and reproducible across sites and cohorts, demonstrating that [^99m^Tc]MDP SPECT in *mdx* mice can be used as a reliable multi-site imaging assay to track muscle injury. Higher muscle uptake of [^99m^Tc]MDP SPECT in *mdx* mice was seen as early as 6 weeks of age and may therefore be an early marker of muscle injury. Although further studies are necessary to support our findings and to leverage the applicability of our approach for studying disease progression and drug effect in the clinical setting, we believe that [^99m^Tc]MDP SPECT may be a useful imaging assay in muscular dystrophy.

## Electronic Supplementary Material

ESM 1 (MP4 5070 kb)
